# Inhibition of IRE1α-driven pro-survival pathways is a promising therapeutic application in acute myeloid leukemia

**DOI:** 10.18632/oncotarget.7702

**Published:** 2016-02-25

**Authors:** Haibo Sun, De-Chen Lin, Xiao Guo, Behzad Kharabi Masouleh, Sigal Gery, Qi Cao, Serhan Alkan, Takayuki Ikezoe, Chie Akiba, Ronald Paquette, Wenwen Chien, Carsten Müller-Tidow, Yang Jing, Konstantin Agelopoulos, Markus Müschen, H. Phillip Koeffler

**Affiliations:** ^1^ Department of Hematology and Oncology, Cedars-Sinai Medical Center, Los Angeles, CA, USA; ^2^ Department of Surgery, University of California San Francisco, San Francisco, CA, USA; ^3^ Department of Oncology, Hematology and Stem Cell Transplantation, RWTH Aachen University Medical School, Aachen, Germany; ^4^ Department of Hematology and Respiratory Medicine, Kochi University, Nankoku, Kochi, Japan; ^5^ Department of Medicine, University of California at Los Angeles, Los Angeles, CA, USA; ^6^ Cancer Science Institute of Singapore, National University of Singapore, Singapore; ^7^ Department of Hematology and Oncology, University Hospital Halle, Halle, Germany; ^8^ Department of Medicine, Hematology and Oncology, University of Muenster, Muenster, Germany; ^9^ Department of Laboratory Medicine, University of California San Francisco, San Francisco, CA, USA

**Keywords:** IRE1, ER stress, XBP1, unfolded protein response, micro RNA

## Abstract

Survival of cancer cells relies on the unfolded protein response (UPR) to resist stress triggered by the accumulation of misfolded proteins within the endoplasmic reticulum (ER). The IRE1α-XBP1 pathway, a key branch of the UPR, is activated in many cancers. Here, we show that the expression of both mature and spliced forms of *XBP1* (*XBP1s*) is up-regulated in acute myeloid leukemia (AML) cell lines and AML patient samples. IRE1α RNase inhibitors [MKC-3946, 2-hydroxy-1-naphthaldehyde (HNA), STF-083010 and toyocamycin] blocked *XBP1* mRNA splicing and exhibited cytotoxicity against AML cells. IRE1α inhibition induced caspase-dependent apoptosis and G1 cell cycle arrest at least partially by regulation of Bcl-2 family proteins, G1 phase controlling proteins (p21^cip1^, p27^kip1^ and cyclin D1), as well as chaperone proteins. Xbp1 deleted murine bone marrow cells were resistant to growth inhibition by IRE1α inhibitors. Combination of HNA with either bortezomib or AS_2_O_3_ was synergistic in AML cytotoxicity associated with induction of p-JNK and reduction of p-PI3K and p-MAPK. Inhibition of IRE1α RNase activity increased expression of many miRs in AML cells including miR-34a. Inhibition of miR-34a conferred cellular resistance to HNA. Our results strongly suggest that targeting IRE1α driven pro-survival pathways represent an exciting therapeutic approach for the treatment of AML.

## INTRODUCTION

Acute myeloid leukemia (AML) is an aggressive hematological malignancy characterized by a small population of self-renewing leukemic stem cells (LSCs) giving rise to a large population of immature leukemic blasts [1–4]. LSCs are relatively insensitive to current therapies [3]. Many AML cells initially respond to treatment; however, relapse is often caused by LSCs that are intrinsically resistant to chemotherapy [5–7]. The overall long-term survival of AML patients remains extremely disappointing at approximately 30% to 50% [1, 8, 9]. Novel therapeutic approaches are clearly needed.

Hematopoietic cells, including LSCs, are exposed to low levels of oxygen in the bone marrow, which may cause accumulation of misfolded proteins in the endoplasmic reticulum (ER), thereby stimulating ER stress and activating the unfolded-protein-response (UPR) pathway [5, 6, 10–12]. ER stress and its UPR are properly compensated in normal marrow hematopoietic cells [13, 14]. In contrast, leukemic cells proliferating in a hostile environment of low oxygen and limited nutrients accumulate misfolded proteins in the ER, causing continuous ER stress with initiation of UPR [15, 16]. Furthermore, leukemic cells produce mutant proteins at a high rate resulting in misfolded proteins [17]. Sustained UPR initiates cellular defense mechanisms rescuing leukemic cells from extreme cellular stress by limiting de novo entry of proteins into the ER which in turn enhances both protein folding capacity and degradation activity [16]. The ability of leukemic cells to handle ER stress may allow them to escape apoptosis and continue their growth [16, 18].

Inositol-requiring enzyme 1 alpha (IRE1α) is one of major ER transmembrane sensors that activates the UPR [19]. IRE1α is evolutionarily conserved in eukaryotes [19]. It has both Ser/Thr protein kinase, as well as endoribonuclease (RNase) activities. Upon activation, IRE1α initiates an unconventional removal of a 26 base intron from the x-box binding protein 1 (XBP1) mRNA, producing an active transcription factor (XBP1s). XBP1s stimulates synthesis of several UPR target genes including ER chaperones, Endoplasmic-Reticulum-Associated protein Degradation (ERAD) components and transcription factors which function to relieve protein misfolding [19, 20]. However, IRE1α acts as a double-edged sword. If restoring ER homeostasis fails, IRE1α represses adaptive responses and initiates apoptosis through Regulated IRE1-Dependent Decay (RIDD) of a large list of substrates which may eventually induces cell death[19, 20]. Caspase-2 (CASP2) is a pro-apoptotic protease required to mediate cellular apoptosis [21]. Up-regulation of CASP2 initiates the intrinsic pathway of apoptosis. During RIDD, IRE1α cleaves and inactivates anti-Casp2 pre-miRNAs (miR-17, miR-34a, miR-96, and miR-125b) resulting in up-regulation of CASP2. Elevated expression of CASP2 helps initiate apoptosis through activating Bid, which causes release of mitochondrial cytochrome c into the cytoplasm [22, 23]. TXNIP is another direct target of miR-17 [24, 25]. TXNIP regulates ER stress-related apoptosis. RIDD increases TXNIP expression through decay of miR-17 [21–23].

The functions of the ER and its associated stress pathways in AML have been studied including recent studies that found approximately 25% of AML samples had detectable *XBP1s*, indicating activation of UPR and an increase of chaperone proteins [26, 27]. Many studies have reported that perturbing the UPR with proteasome inhibitors, such as bortezomib, can enhance apoptosis of AML cells [28–32]. In this study, we explored whether AML cells have activated IRE1α which can be therapeutically targeted.

## RESULTS

### XBP1 and XBP1s are up-regulated in AML

**Figure 1:**
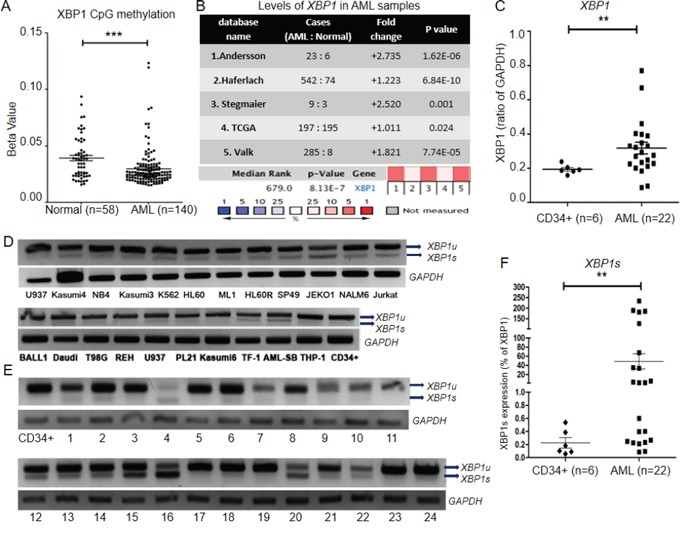
*XBP1* and *XBP1s* are up-regulated in AML **A.** The methylation status of the CpG islands of XBP1 in normal donors (n=58) and AML samples (n=140) was analyzed using TCGA level 3 database. The p-values were calculated by student t test. **B.** 5 publicly available microarray databases showed *XBP1* was highly expressed in AML samples compared with normal BM samples. 1. Andersson Leukemia [84]; 2. Haferlach [85]; 3. Stegmaier [86]; 4. TCGA [87]; 5. Valk [88]. The rank for a gene is the median rank for that gene across each of the analyses. The p-value refers to the median-ranked analysis. **C.** QRT-PCR analysis of AML blast cells from patients (n=22) compared with normal human CD34+ cells (n=6) showed significant up-regulation of XBP1, using GAPDH as an internal control (p<0.01). **D, E.** RT-PCR and gel electrophoresis identified *XBP1s* activation in human leukemia cell lines (D) and samples from normal (CD34+) and AML blast cells from patients (1-24) (E). **F.** QRT-PCR analysis of *XBP1s* expression in AML blast samples from patients (n=22) and normal human CD34+ cells (n=6). Figures are representative example of 3 replicates. Data represent mean ± SD. *XBP1s*, spliced XBP1.

IRE1 signaling pathway through XBP1 and XBP1s is strongly linked with ER stress and UPR [33]. To examine if XBP1 has a crucial role in AML, we first analyzed the AML methylation database (27k Illumina methylation version) from TCGA. Compared with normal samples, *XBP1* was highly hypomethylated on its CpG island in AML cases (Figure 1A). Consistent with the methylation status, *XBP1* expression was significantly up-regulated in AML cases [5 previously published microarray databases (Figure 1B) and our QRT-PCR results (Figure 1C)]. A combination analysis of the 5 published databases showed that *XBP1* ranked No. 679th of the most highly expressed genes in AML (Figure 1B). Results were calculated by online analysis engine Oncomine (https://www.oncomine.org/resource/login.html). Interestingly, *XBP1s* was detectable in 85% (22 of 26) of the leukemia cell lines and 71% (17 of 24) of AML patient samples (Figures 1D, 1E). Normal purified CD34+ myeloid stem cells did not have detectable *XBP1s* (Figure 1E). *XBP1s* was also significantly elevated in AML samples from patients compared to CD34+ normal myeloid stem cells (p=0.0043, n=28) as measured by QRT-PCR (Figure 1F). To investigate correlations between *XBP1* expression and AML clinical features, we first performed statistical analysis to correlate the expression of *XBP1/XBP1S* with French-American-British (FAB) subtypes in our own dataset (Table S2 and Figure 1C, 1E, 1F). However, probably due to the limited numbers of cases, we did not observe a significant association between *XBP1/XBP1s* and FAB subtypes among the 24 AML samples (data not shown). We next performed similar statistical analysis using TCGA AML dataset. Since *XBP1s* was not discernable from total *XBP1* in the dataset, we only tested total *XBP1* level. Interestingly, *XBP1* expression was significantly increased in FAB M3 subgroup compared with M0, M1 and M2 but significantly decreased in M4-M7 subgroup (Figure S1). The biological significance of these correlations requires further investigations.

### IRE1α RNase inhibitors blocked splicing of XBP1 mRNA and exhibited cytotoxicity against AML cells

**Figure 2:**
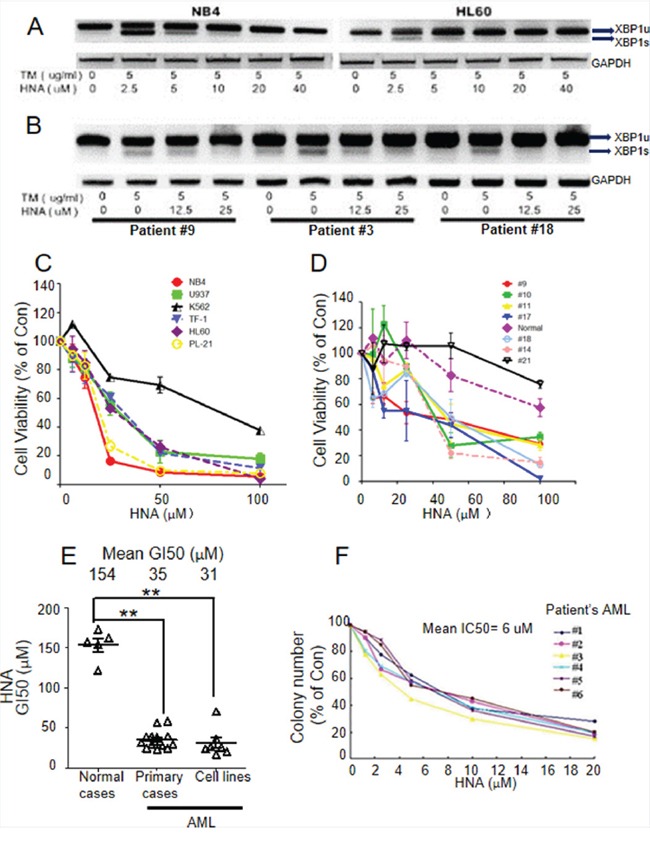
HNA inhibits *XBP1s* and causes cytotoxicity of AML cells **A, B.** HNA inhibited *XBP1s* expression induced by tunicamycin (TM) in human AML cells lines (A) and AML blast cells from patients (B). Cells (10^6^) were incubated with indicated concentrations of either TM or TM and 2-hydroxy-1-naphthaldehyde (HNA) in 6-well plates for 6 h. RNA was isolated and RT-PCR was performed to examine *XBP1u* and *XBP1s* expression by gel electrophoresis. **C, D.** Cell viability analysis examined IRE1 inhibitor induced cytotoxicity of human AML cell lines (C) and AML blast samples from normal and AML patients (D). Cells (10,000) were added into 96-well plates followed by exposure to various concentrations of HNA. Cell viability (MTT assay) was examined 72 h later. **E.** Concentration for 50% of maximal inhibition of cell proliferation (GI_50_) of HNA for 15 AML patient samples and 7 AML cell lines (NB4, U937, K-562, TF-1, HL-60, PL-21 and THP-1) calculated from data shown in Figures 2C and 2D. “Con”, untreated control. The GI_50s_ were calculated by Graphpad software. **F.** Soft agar clonogenic assays of 6 AML patient samples exposed to HNA. Figures are representative example of 3 replicates. GI_50s_ were calculated. Data represent mean ± SD, n=3.

Recently, a novel small-molecule RNase inhibitor of IRE1 (MKC-3946) was noted to have potent anti-proliferative activity in multiple myeloma (MM) [34]. The compound was found to be very unstable *in vitro*; however, one of the two major hydrolyzed precursors, A-106 (2-hydroxy-1-naphthaldehyde, HNA) retained the IRE1α RNase inhibitory activity [35]. Tunicamycin (TM) induces ER stress and *XBP1* splicing in many cells [36]. Following TM treatment, increased expression of *XBP1s* mRNA and decreased *XBP1u* (unspliced, transcriptional inactive form of XBP1) were observed in 293T and K562 myeloid leukemia cells (Figure S2A). Compared with MKC-3946, HNA showed either the same or more potent ability to inhibit the activity of IRE1α to cleave XBP1 into the active XBP1s after TM induced activation of NB4 cells (Figure S2B). STF-083010 is a newly developed IRE1α endonuclease specific inhibitor which has shown cytotoxic activity against human multiple myeloma [37, 38]. Treatment of AML cells with increasing drug dosage showed slightly enhanced potency of HNA compared to STF-083010 (Figures S3A-D). HNA dose-dependently inhibited XBP1s expression induced by TM in AML cell lines and AML patient samples (Figures 2A-2C). HNA significantly decreased cellular viability of both AML cell lines (mean GI_50_=31 μM, n=8) and AML patient samples (mean GI_50_=35 μM, n=18) compared to untreated patient samples (mean GI_50_=154 μM, n=5, Figures 2C-2E). Importantly, HNA caused a significant inhibition (mean GI_50_=6 μM, n=6) of clonogenic growth in soft agar of AML cells from patients (Figure 2F). In contrast, HNA had very low toxicity against normal human marrow mononuclear cells (mean GI_50_=123 μM, n=4) (Figure 2E). We conducted western blotting assay on BALL1, REH and K562 cell lines, and confirmed that the XBP1s protein levels were correlated with their mRNA levels. Specifically, K562 cells showed expression of both XBP1s mRNA and protein, whereas BALL1 and REH cells expressed neither mRNA nor protein of XBP1s (Figures 1D and S2D). Furthermore, we confirmed that the ER stress inducer thapsigargin successively induced XBP1s expression (Figure S2D).

### IRE1α induced apoptosis and G1 cell cycle arrest in AML

**Figure 3:**
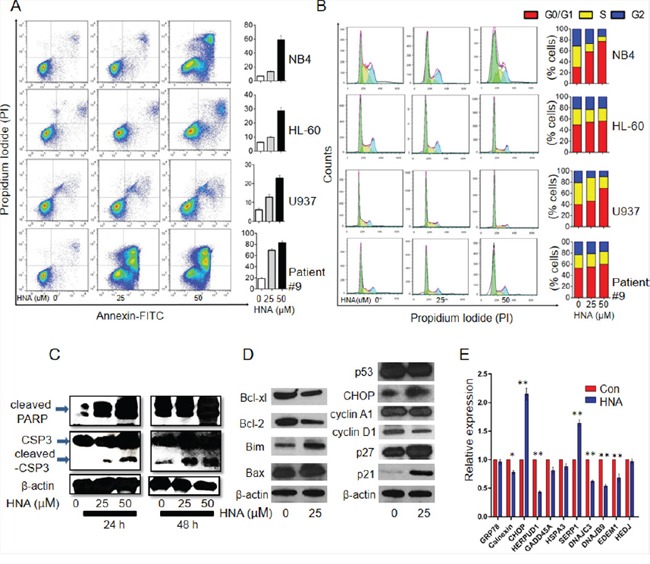
IRE1α inhibition induced apoptosis and G1 cell cycle arrest in AML cells **A.** AML cells (NB4, HL-60, U937 and AML patient sample #9) were treated with HNA (25μM, 50μM) for 24 h and Annexin/PI assays were conducted to evaluate HNA induced apoptosis. Right side bar graphs show percent apoptotic cells (positive Annexin V + PI) in each treatment group. **B.** AML cells (NB4, HL-60, U937 and AML patient sample #9) were treated with HNA (25μM and 50μM) for 24 h and stained with PI. Cell cycle was analyzed by Flowjo software. Right panel, bar graphs displayed cell cycle phase distribution in each treatment group. Cells cultured with diluent were used as control (Con). **C.** NB4 cells were treated with HNA (25 μM, 50 μM) for either 24 or 48 h and western blotting evaluated expression of cleaved PARP and cleaved caspase-3. β-actin was used as loading control. **D, E.** NB4 cells were treated with HNA (25 μM, 48 h) and expression of Bcl-2 family and cell cycle associated proteins were evaluated by western blotting (β-actin, loading control) (D); mRNA levels of chaperone genes were measured by QRT-PCR (E). Relative expression of each gene was normalized to GAPDH mRNA; and for each gene, control levels were considered to be 1.0. Figures (A, B, E) are representative example of 3 replicates. Data represent mean ± SD, n=3.

Inhibition of UPR blocks pro-survival pathways is implicated in apoptosis and cell-cycle arrest in several model systems [39]. In a dose-dependent manner, HNA treatment of AML cell lines and primary samples significantly increased the percent annexin V positive cells (Figures 3A, S4), increased the percent of cells in G1 of the cell cycle (Figure 3B), increased cleaved PARP and caspase-3 (Figure 3C), down-regulated Bcl-2 pro-survival family members (Bcl-2 and Bcl-xl), up-regulated the pro-apoptotic protein, Bim (Figures 3D, S5), increased G1 phase regulators (p21^cip1^, p27^kip1^), and decreased level of cyclin D1 (Figure 3D). Chaperone protein CHOP is up-regulated by ER stress and enhances ER stress induced apoptosis. However, other chaperone proteins such as Calnexin, HERPUD1, DNAJC3, DNAJB9 and EDEM are activated by UPR which is beneficial for cell survival during ER stress [40, 41]. Our results showed HNA treatment increased CHOP mRNA and protein (a chaperone molecule) (Figures 3D, 3E, S5). In contrast, several other chaperone genes *Calnexin*, *HERPUD1*, *DNAJC3*, *DNAJB9* and *EDEM* were significantly down-regulated by HNA (Figure 3E). These results indicate that IRE1α inhibition induced cell death by blockage of pro-survival UPR pathways and enhancement of pro-apoptotic pathways.

### Absence of Xbp1 in murine myeloid cells produced resistance to Ire1α inhibitors

**Figure 4:**
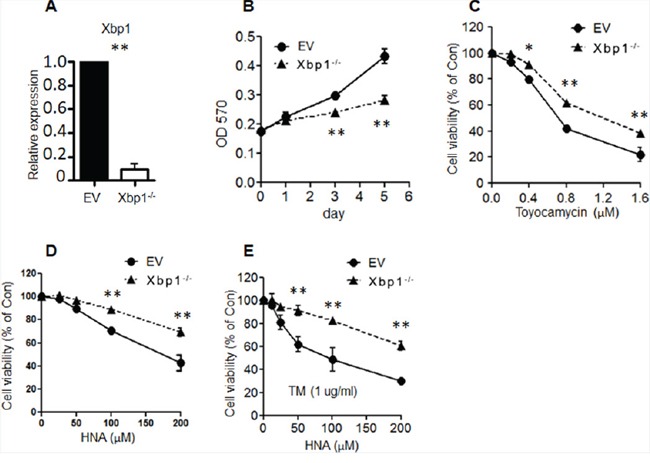
Knock-out Xbp1 induced myeloid cell resistance to IRE1 inhibitors **A.** Xbp1 ^flox/flox^ murine bone marrow cells were infected with a retroviral vector that expressed either the Cre recombinase or empty vector (EV); these cells were stably selected with G418 followed by addition of 4-OHT (1μM) for two days to obtain either Cre-mediated Xbp1 knock-out (Xbp1^−/−^) or empty vector (EV) Xbp1^fl/fl^ myeloid cells. QRT-PCR was performed to measure knock-out effenciency of Xbp1. **B.** Xbp1^−/−^ and EV marrow cells (1,000) were seeded into 96-well plates, and cell proliferation was measured on days 1, 3 and 5. (MTT assay) (n=3). **C-E.** Xbp1^−/−^ and EV marrow cells were (1,000) seeded into 96-well plates and followed by treatment with increasing concentrations of IRE1 inhibitor [Toyocamycin alone (C); HNA alone (D); HNA or TM plus HNA (E)]. After 72h, cell viability was measured (MTT assay). Data represent mean ± SD, n=3.

To assess whether Xbp1 is a major driver of Ire1α signaling pathway, we generated a model based on bone marrow progenitor cells from mice carrying a floxP-flanked allele of *Xbp1* (*Xbp1^flox/flox^*). After Cre activation, more than 92% of *Xbp1* was confirmed to be deleted in the floxed cells as evidenced by QRT-PCR (Figure 4A). Ire1α-Xbp1 *in vivo* inhibition effect was confirmed as HNA decreased TM induced *Xbp1s* levels in bone marrow cells of mice (Figure S6). Myeloid cells (94 % CD34+) with deleted *Xbp1* showed slower proliferation (Figure 4B) and increased resistance to both IRE1α inhibitors (toyocamycin and HNA) (Figures 4C, 4D). Treatment with TM (1 mg/ml) produced negligible cytotoxicity to the murine BM cells (Figure S8B). Combination of TM (1 mg/ml) with HNA, increased cell viability in *Xbp1^−/−^* cells compared to vector control cells (Figures 4E), suggesting that Ire1α induced UPR is at least partially reliant on Xbp1.

### Combination of HNA with either bortezomib or AS_2_O_3_ synergistically inhibited growth of AML cells

**Figure 5:**
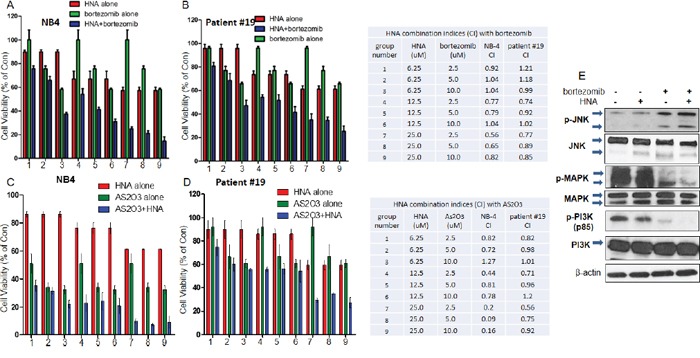
Combination of HNA with either bortezomib or AS_2_O_3_ NB4 **A, C.** or #19 primary AML blast cells **B, D.** were seeded (10,000) in 96-well plates and treated with HNA (0, 6.25, 12.5 and 25 μM) and/or bortezomib (0, 2.5, 5, 10 μM) (A, B) or HNA and/or AS_2_O_3_ (0, 2.5, 5, 10 μM) (C, D) for 72 h; and cell viability was measured (MTT assay). Data are presented as percentage of diluent treatment control (Con). Data represent mean ± SD, n=3. CI (<1, synergistic; =1, additive; >1, antagonistic). **E.** NB4 cells were treated with HNA (25 μM) and/or botezomib (5 μM) for 48 h, and expression of total and phosphorylated (p-) JNK, MAPK and PI3K were evaluated by western blotting (β-actin, loading control).

Bortezomib is a potent 26S proteasome inhibitor which induces terminal UPR and apoptosis in many cells [42–45]. AS_2_O_3_ is a leading therapy for treatment of acute promyelocytic leukemia (APL) [46] and has recently been shown to induce ER stress in cancer cells including leukemia cells [47, 48]. Both drugs induced apoptosis associated with p-JNK activation in cancer cells [49, 50]. We hypothesized that the combination of an IRE1α RNase inhibitor with either bortezomib or AS_2_O_3_, will enhance the inhibition of proliferation of AML cells. Indeed, various concentrations of either drug combined with HNA synergistically inhibited growth of both NB4 cells and an AML patient sample #19 (Figures 5A-5D). Also, bortezomib induced expression of p-JNK, as well as reduced levels of p-MAPK and p-PI3K in NB4 cells. (Figure 5E).

### Inhibition of IRE1α RNase activity increased the expression of selected miRs in AML

**Figure 6:**
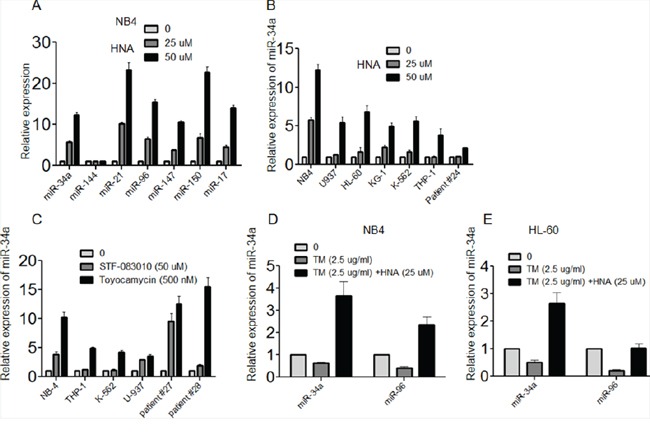
Inhibition of IRE1α increased expression of miRs in AML cells **A.** NB4 cells were treated with HNA (0, 25, 50 μM) for 24h and expression of pre-miR- 34, -144, -21, -96, -147 and -150 was measured by QRT-PCR. Cells without HNA were used as control. **B.** QRT-PCR analysis of pre-miR-34a expression level in NB4, U937, HL-60, KG-1, K-562 and THP-1 AML cell lines and primary AML blast sample #24 upon HNA (25 μM) treatment for 24 h. **C.** QRT-PCR analysis of pre-miR-34a expression level in NB4, THP-1, K-562 and U937 AML cell lines and primary AML blast cell samples #27 and #28 after exposure to either STF-083010 (50 μM) or Toyocamycin (500 nM) for 24 h. **D, E.** NB4 or HL-60 cells were treated with either TM (2.5 μg/ml) alone or TM and HNA (25 μM) for 12 h; expression levels of pre-miR-34a and miR-96 were measured by QRT-PCR. Relative expression of each gene was normalized to GAPDH. Data represent mean ± SD, n=3.

Recent studies discovered that IRE1α could cleave 4 anti-Casp2 pre-miRNAs (miR-17, -34a, -96, and -125b), resulting in activation of CASP2 in mouse embryonic fibroblasts (MEFs) [22, 23]. To assess whether the cleavage of miRs occurs in AML, we examined expression levels of pre-miRs upon treatment of NB4 AML cells with IRE1α inhibitors. The pre-miRs -17, -21, -34a, -147 and -150 were dramatically increased after exposure of the AML cells to IRE1α inhibitor (HNA, 25, 50 μM) in a dose-dependent manner (Figures 6A, 6B). Also, U937, HL-60, KG-1 and K562 cells responded to the IRE1 inhibitor (HNA) by increasing the levels of miR-34a (Figure 6B). Furthermore, other IRE1α inhibitors (STF-083010, 50 μM; Toyocamycin, 500 nM) dramatically increased miR-34a in AML cells (NB4, THP-1, K562, U-937, patients #27 and #28, Figure 6C). In contrast, exposure to TM (2.5 μg/ml, 12 h), a known IRE1 enhancer, slightly inhibited levels of miR-34a and miR-96 even in the presence of HNA (25 μM; 12 h) (Figure 6D). Actinomycin D, a DNA transcription inhibitor inhibited HNA induced miR-34a activation (Figure S7). In the presence of TM (1-4 μg/ml; 72 h), murine myeloid cells with deletion of Xbp1 (following Cre activation) had no change in either the expression of miR -34a, -96, -147 and -150 (Figure S8A) or in cell growth (Figure S8B). Murine BM cells treated with HNA (25, 50 μM; 12 h) also displayed a dose-dependent increase of pre-miRs which was independent of Xbp1 (Figure S8C), suggesting that this regulation might be directly through IRE1-driven RIDD.

### miR-34a triggered sensitivity of IRE1 inhibitor *in vitro*

**Figure 7:**
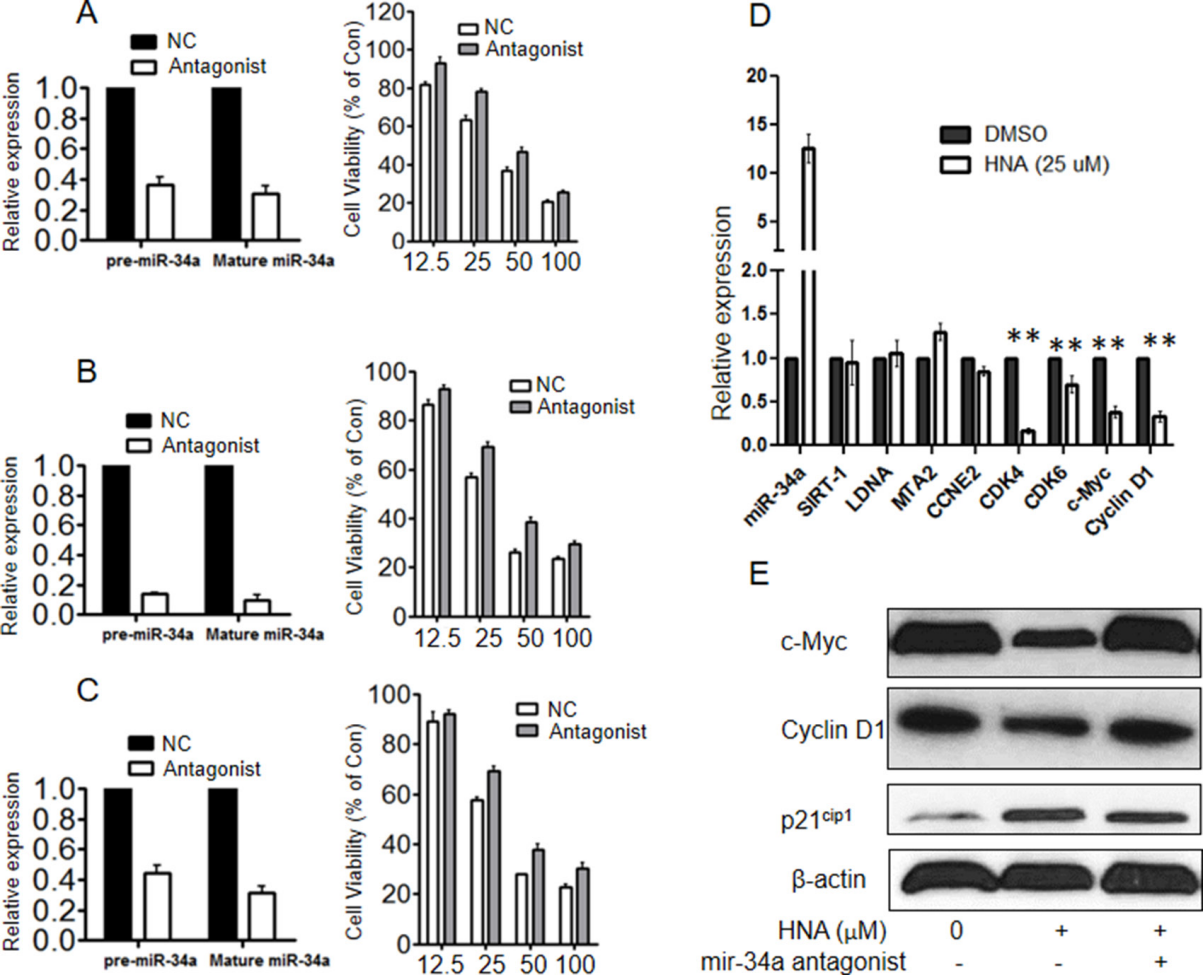
Blockade of miR-34a decreased sensitivity of IRE1 inhibitor *in vitro* **A-C.** miR-34a small RNA antagonist or control small RNA were transiently transfected and knock-down efficiencies of either pre-miR-34a or mature miR-34a were evaluated by QRT-PCR in K562 (A, left side), NB4 (B, left side) and U937 (C, left side). At 24 h after transfection, cells (10,000) were seeded into 96-well plates followed by treatment with HNA (0, 12.5, 25, 50 μM) for 72h, and cell viability was measured (MTT assay) (A-C, right side). **D.** NB4 cells were treated with HNA (25 μM, 24 h) or diluent control. mRNA expression levels of pre-miR-34a, SRRT-1, LDNA, MTA2, CCNE2, CDK4, CDK6, c-Myc and cyclin D1 were measured by QRT-PCR. Relative expression of each gene was normalized to GAPDH. **E.** At 48 h after transfection of miR-34a antagonist or control siRNA, NB4 cells were treated with HNA (25 μM, 48 h) or diluent control, and protein expressions of c-Myc, cyclin D1 and p21^cip1^ were evaluated by western blotting (β-actin as loading control). Data represent mean ± SD, n=3.

Small RNA antagonist against miR-34a was transiently transfected into 3 AML cell lines (K562, NB4, U937), and cell viability was examined after treatment with HNA (12.5-100 μM). Knockdown efficiency of miR-34a by the antagonist was validated both at the pre- and mature miR level by QRT-PCR (Figures 7A-7C, left panels). Inhibition of miR-34a conferred modest survival advantage compared to HNA alone in these AML cells (Figures 7A-7C, right panels). In addition, HNA inhibited transcriptional levels of several targeted genes of miR-34a (c-Myc, cyclin D1, CDK4) in NB4 cells (Figure 7D); Also in the AML cells, the miR-34a antagonist restored protein expression levels of c-Myc and cyclin D1 proteins, these had been inhibited in their expression in the presence of HNA alone. (Figure 7E).

## DISCUSSION

The UPR is a defense mechanism activated by cells during stressful conditions in response to an accumulation of misfolded proteins in the ER [51]. Cancer cells are usually exposed to many stressful environments (e.g. hypoxia, nutrient starvation, oxidative stress and other metabolic dysregulation) resulting in continued ER stress. Furthermore, genomic mutations can also lead to accumulation of misfolded proteins. The UPR is often activated to help cancer cells escape from ER stress-induced cell death [52]. Reactive oxygen species (ROS) in AML cells can also stimulate ER stress and the UPR [11, 53, 54]. Studies have particularly focused on the functional roles of UPR proteins in multiple myeloma cells because chronic ER stress occurs in these cells [37, 55, 56]. Activation of the unfolded protein response including XBP1 splicing has been noted in AML samples [26, 27]. In our study, we confirmed that one of the major branches of the UPR (IRE1-XBP1s) is frequently activated in AML cell lines and AML patient samples, suggesting that targeting the UPR may be a promising adjunctive approach for treatment of AML.

XBP1 and its spliced form XBP1s have been reported to be upregulated in several types of cancers, and blockage of the IRE1/XBP1 pathway is considered as a promising therapeutic option [55, 57–61]. XBP1 is one of the most well studied genes modulated by IRE1α, which has important roles in the regulation of cell survival and UPR as a downstream target of IRE1α [55, 62–65]. However, XBP1 is not the only factor in response to IRE1α inhibition. In fact, as an RNase, IRE1α recognizes and cleaves a consensus element, CUGCAG, in target RNAs upon cellular stresses [66]. Therefore, IRE1α has the capacity to target directly a number of RNAs (both coding and noncoding) and regulate their expression levels through Regulated IRE1-Dependent Decay (RIDD). For example, in a recent study using MEF cells, IRE1α was found to cleave four anti-CASP2 miRs (miR-17, -34, -96 and -125) [22, 23]. Based on our bioinformatic analysis of miRs and RNA microarray results, we found that many pre-miRs with IRE1α cutting consensus motif were up-regulated upon HNA treatment. We focused on 5 of these candidate miRs and QRT-PCR results demonstrated that they were induced in the presence of IRE1α inhibitors (Figure 6). Together, these results suggest that the anti-neoplastic effects of IRE1α inhibitors are the results of the enhanced nuclease activity that generates not only XBP1s, but also a number of other RNAs, including miRNAs.

We particularly focused on miR34a. Recent studies indicate that miR-34a is a tumor suppressor [67]. Down-regulation of miR-34a causes resistance to chemotherapy [67–69]. Targets of miR-34a include caspase-2, c-Myc, Bcl-2, cyclin D1, MET and SIRT1. miR-34a dependent inhibition of SIRT1 can increase acetylation and activation of p53 resulting in up-regulation of p21 and PUMA [70]. By post-transcriptional blockade of these genes, miR-34a suppresses migration and induces apoptosis, G1 cycle arrest and senescence in cancer cells [71, 72]. Our study showed that IRE1α inhibitors significantly increased pre- and mature miR-34a mRNA levels, associated with inhibition of *CDK4*, *c-Myc*, *Bcl-2* and *Cyclin D1* and induction of *p21^cip1^* and *p27^kip1^* in AML cells. Silencing of miR-34a by small RNA antagonist significantly induced resistance of AML cells to IRE1α inhibitors and restored levels of miR-34a targeted oncogenes such as c-Myc and Cyclin D1. These results indicate that miR-34a plays an important role in IRE1α-dependent UPR in AML. Through cleavage of miR-34a, IRE1α decreases miR-34a induced apoptosis and helps AML cells to escape death.

Bortezomib is a proteasome inhibitor which has been approved by FDA as the first example of UPR-modulating regimen for the treatment of Multiple Myeloma [73]. Recently, bortezomib was also approved for treatment of mantle cell lymphoma [74]. In the context of AML, several clinical studies have shown that bortezomib has potent anti-neoplastic activity, and the mechanisms include induction of apoptosis and transcriptional inactivation of several important AML-drivers such as DNA methyltransferases (DNMTs) and receptor tyrosine kinases (RTKs) [29, 38, 75, 76]. Moreover, bortezomib has shown greater therapeutic value when applied in combination with traditional cytotoxic chemotherapies to treat AML [31, 77, 78]. Therefore, although not yet approved by FDA, bortezomib has demonstrated its promising therapeutic merit and is considered as one of the candidate drugs for the treatment of AML. On the other hand, several studies have shown that IRE1α inhibitors in combination with bortezomib strongly impaired the growth of multiple myeloma cells both *in vitro* and *in vivo* [37, 55]. Mechanistically, IRE1α inhibition can overcome the tumor cytoprotective effects conferred by bortezomib-induced UPR via activation of XBP1s [55]. In the present study, we also showed that combined addition of HNA with bortezomib synergistically increased apoptosis of AML cells associated with the up-regulation of CHOP and p-JNK. Therefore, we believe that our results will provide useful information to the investigators who are interested in developing bortezomib for the treatment of AML.

Treatment of acute promyelocytic leukemia (APL) with AS_2_O_3_ has unique favorable cure rates [79], and the major mechanism of action is through the degradation of PML-RARα, the driver of APL [80]. Notably, AS_2_O_3_ has been reported to enhance UPR by increasing the expression of GRP78, CHOP, phosphorylated eIF2α and ATF4 [81, 82]. In our study, the combination of HNA and AS_2_O_3_ synergistically increased cell apoptosis of the APL cells NB4, which might be due to corroborative inhibition of different branches of the UPR pathway. In the previous clinical trials, QT interval prolongation and APL differentiation syndrome are the most serious side-effects of AS_2_O_3_ treatment [83]. The combined use of HNA and AS_2_O_3_ may improve the toxicity profile.

In conclusion, we found that *XBP1* and its spliced form (*XBP1s*) are often increased in AML. Inhibition of IRE1α RNase activity by small molecules inhibited AML cell growth. This inhibition of proliferation probably occurred by inducing ER stress by blocking the compensatory pathways including the prevention of cleavage of tumor suppressor miRs (e.g. miR-34a) which may augment the anti-proliferative effect of IRE1α inhibitors. Therefore, compounds which inhibit activation of IRE1α represent a novel pathway for cell kill and may be a useful compliment to chemotherapy.

## MATERIALS AND METHODS

Information of reagents and vendors, and all methods are described in the Supplemental Methods.

## SUPPLEMENTARY FIGURES AND TABLES





## Abbreviations

AML: acute myeloid leukemia
AS_2_O_3_: arsenic trioxide
UPR: unfolded protein response
HNA: 2-hydroxy-1-naphthaldehyde
LSCs: leukemic stem cells
HSCs: Multipotent hematopoietic stem cells
ER: endoplasmic reticulum
IRE1α: Inositol-requiring enzyme 1 alpha
ERAD: Endoplasmic-reticulum-associated protein degradation
RIDD: Regulated IRE1-Dependent Decay
MTT: 3-(45-dimethylthiazol-2-yl)-25-diphenyltetrazolium bromide
TCGA: The Cancer Genome Atlas
n: denotes the number of independent experiments
HL60R: HL60 derived all-trans retinoic acid (ATRA) resistant cell line.
