# Correction: Inhibition of IRE1α-driven pro-survival pathways is a promising therapeutic application in acute myeloid leukemia

**DOI:** 10.18632/oncotarget.20707

**Published:** 2017-09-08

**Authors:** Haibo Sun, De-Chen Lin, Xiao Guo, Behzad Kharabi Masouleh, Sigal Gery, Qi Cao, Serhan Alkan, Takayuki Ikezoe, Chie Akiba, Ronald Paquette, Wenwen Chien, Carsten Müller-Tidow, Yang Jing, Konstantin Agelopoulos, Markus Müschen, H. Phillip Koeffler

**Present**: The grant information is incomplete.

**Correct**: Additional grant information is shown below.

Original article: Oncotarget. 2016; 7:18736-18749. https://doi.org/10.18632/oncotarget.7702

**GRANT SUPPORT**

National Natural Science Foundation of China (NSFC, 31401594, to X.G).

