# Supplementary material for: Inhibition of IRE1α-driven pro-survival pathways is a promising therapeutic application in acute myeloid leukemia
Source: Oncotarget. 2016 Feb 25;7(14):18736–49. doi: 10.18632/oncotarget.7702 (PMC4951325; doi:10.18632/oncotarget.7702)
Supplement: Supplementary file 1 [file oncotarget-07-18736-s001.pdf]

SUPPLEMENTARY DATA

Reagents and vendors

MKC-3946(3-Ethoxy-5,6-dibromosalicylaldehyde), STF-083010 (N-[(2-Hydroxy-1-naphthalenyl)methylene]-2-thiophenesulfonamide), HNA (A106, 2-Hydroxy-1-naphthaldehyde), propidium iodide (PI), tunicamycin (TM), thapsigargin, polyethylenimine (PEI), 4-hydroxy tamoxifen (4-OHT) and 3-(4,5-dimethylthiazol-2-yl)-2,5-diphenyltetrazolium bromide (MTT) were purchased from Sigma-Aldrich (St. Louis, MO); Bortezomib, As_2O_3 and 17-allylamino-17-demethoxygeldanamycin (17-AAG) were obtained from Selleck Chemicals (Houston, TX); recombinant human stem cell factor (SCF), granulocyte-colony stimulating factor (G-CSF), Flt3-ligand and thrombopoietin (TPO); murine stem cell factor (SCF), interleukin-3 (IL-3) and interleukin-6 (IL-6) from PeproTech (London, UK); CD34 microBead kit, Miltenyi Biotec Inc (Auburn, CA); Ficoll-paque plus from GE Healthcare (Piscataway, NJ); Roswell Park Memorial Institute medium (RPMI-1640) from Invitrogen (Carlsbad, CA); Annexin V/PI double staining kit, BD Biosciences (San Jose, CA); Total RNA extraction kit, miScript II RT kit and miScript hiSpec buffer from Qiagen (Valencia, CA); qScript™ cDNA synthesis kit and QPCR sybr green master mix from Quanta Biosciences (Gaithersburg, MD); RIPA Lysis Buffer, nitrocellulose (NC) and PVDF membranes from Millipore (Billerica, MA); protease inhibitor cocktail and phosphatase inhibitor cocktail from Roche Applied Science (Indianapolis, IN); BCA assay kit and superSignal west dura chemiluminescent substrate, Thermo Scientific (Rockford, IL); RetroNectin, from Clontech Inc (Mountain View, CA); miR-34a antagomir and antagomir negative control, from GenePhama (Shanghai, China); Amaxa nucleofector kit from LonZa (Atlanta, GA).

Antibodies

Antibodies against CHOP (D46F1), Bcl-2, Bim (C34C5), Bax (D2E11), PARP (46D11), pJNK (81E11), pMAPK (197G2), MAPK (137F5) and PI3K (19H8) were purchased from Cell Signaling (Beverly, MA, USA). Antibody against β-actin was purchased from Sigma (St Louis, MO, USA). pPI3K (ab182651) was purchased from Abcam (Cambridge, MA, USA). CSP3 (H-277), Bcl-XL (H-5), p53 (FL-293), Cyclin A1 (H-230), Cyclin D1 (A-12), $p27^{kip1}$(C-19), $p21^{cip1}$(C-19) and JNK (FL) were purchased from Santa Cruz Biotechnologies (Dallas, TX, USA).

Primary samples and cell lines

Human cord blood samples were collected from healthy volunteers with IRB approved, and mononuclear cells were purified by Ficoll-Paque Plus (GE Healthcare). AML primary samples were obtained from University of California Los Angeles, Cedars Sinai Medical Center, Kochi University and University of Muenster. The French-American-British (FAB) classification and characteristics of AML patients are shown in Table S1. Selected samples of mononuclear cells were enriched for CD34+ cells (95% purity) using Microbead kit. Primary cells were maintained in RPMI-1640 with GlutaMAX containing 20% fetal bovine serum (FBS), penicillin (100 IU/ml) and streptomycin (100 μg/ml), G-CSF, SCF, Flt3-ligand and TPO (50ng/ml each). For murine hematopoietic cells, young age-matched mice were sacrificed, and bone marrow (BM) cells were collected from femurs and tibias. Cells were incubated in red blood cell lysis buffer, washed with phosphate buffered saline (PBS) and cultured in Iscove's Modified Dulbecco's Media (IMDM) containing 20% FBS, plus murine SCF (50 ng/ml), IL3 (10 ng/ml) and IL6 (25 ng/ml) to expand myeloid cells. To test the function of XBP1, bone marrow cells were isolated from $Xbp1^{fl/fl}$ mice, [33] CD34+ cells were purified, and cultured in IMDM culture medium with cytokines as described above. $Xbp1^{fl/fl}$ myeloid cells were subsequently transduced with 4-Hydroxytamoxifen (4-OHT) inducible Cre. Cre-mediated deletion of Xbp1 was accomplished by treatment of these cells with 4-OHT (1μM) for two days.

Cell proliferation, viability assay and examination for synergy

Cell proliferation and viability were examined using MTT assay. For each assay, various number of cells (1,000 for cell proliferation and 10,000 for cell viability assays) were seeded in 96-well plates, followed by either vehicle (DMSO) or increasing concentrations of drug. For detection of relative numbers of living cells, 10 μl of MTT (5 mg/ml) was added to each well, placed in an incubator for four hours, followed by centrifugation (1,000 rpm, 5 min); 100 μl of supernatant media from each well were carefully removed and 100 μl of SDS buffer (20% in water) was added to dissolve the crystals. Results were further read on spectrophotometer machine at 570 nM wavelength. Half maximal inhibitory concentration (IC_{50}) was calculated using the GraphPad Prism 5 (GraphPad Inc., San Diego, CA). Synergy of combination of two drugs was determined

using the CalcuSyn software (BioSoft, Cambridge, UK). The extent of drug interaction between the two drugs was determined using the combination index (CI) for mutually exclusive drugs. Different CI values were obtained when solving the equation for different concentrations of drugs. A CI of 1 indicates an additive effect, whereas a CI of <1 denotes synergy. All experiments were repeated at least three times.

Apoptosis and cell cycle analysis

5×10^5 cells were seeded in 6-well plates followed by culture with either diluent control (DMSO) or various concentrations of experimental drugs. Cells were collected by centrifugation and washed with PBS. Apoptosis was determined by Annexin V/PI staining (BD Biosciences) according to manufacturer's instructions. Cell cycle analyses were performed by propidium iodide staining (Sigma-Aldrich) for DNA content and flow cytometric analysis. All flow cytometry data were analyzed using flowJo software (Tree Star, Ashland, OR).

PCR

Total RNA from cells was extracted using RNeasy isolation kit (Qiagen). Evaluation of relative expression levels of unspliced form of XBP1 (XBP1u)/XBP1s by RT-PCR and electrophoresis was done as previously described.[34] Primers for RT-PCR are listed in Table S2. For Quantitative Real-Time PCR (QRT-PCR), cDNA was generated using qScript™ cDNA Synthesis Kit (Quanta Biosciences). QRT-PCR was performed on CFX96 qPCR System (Biorad Inc, Hercules, CA). Expression of each gene was normalized to GAPDH as a reference. The conditions for all quantitative RT- PCR reactions are as follows: 2 minutes at 95°C followed by 15 seconds at 95°C and 30 seconds at 60°C for 40 cycles. Primers for QRT-PCR are listed in Table S2.

Western blotting

Proteins were lysed from cells using RIPA Lysis Buffer (Millipore, Billerica, MA) supplemented with protease inhibitor cocktail (Roche) and phosphatase inhibitor cocktail (Roche). Protein concentrations were determined by BCA assay (Thermo Scientific). Protein samples were resolved by sodium dodecyl sulfate polyacrylamide gel (SDS-PAGE) electrophoresis, transferred to either 0.45 μM nitrocellulose (NC) or 0.2 μM PVDF membranes (Millipore), and membranes were blocked for 1 hr with TBS containing 5 % non-fat dry milk and 0.1% Tween-20. After blocking, membranes were incubated with primary antibodies at 4 °C overnight. The following day, the membranes were washed with TBST and incubated with a secondary antibody-HRP conjugate at room temperature for one hour. After washing, membranes were visualized with ECL plus Substrates (Thermo Scientific). The antibodies used for Western blotting are listed in supplemental materials.

Retroviral transduction

Retroviral constructs and the corresponding empty vector controls were packaged in Platinum-E (Plat-E) cells using PEI transfection method. 8 μg of plasmid was incubated with 24 μl of PEI reagent (1μg/μl) in 400 μl Opti-MEM media (Invitrogen) for 20 min. The mixture was placed on the Plat-E cells in 10 cm culture dishes. The virus supernatants were harvested 24 h and 48 h later. Viral supernatants from 2 collections were combined, filtered through a 0.45 μm filter, and loaded on RetroNectin (Clontech) coated non-tissue 6-well plates, and 1×10^6 cells/well were transduced following the manufacturer's instructions.

Data base and statistical analysis

Methylation of *XBP1* (AML versus Normal) was analyzed using level 3 data from the TCGA Database. The beta values are continuous and range from 0 (unmethylated) to 1 (completely methylated)[35]. XBP1 mRNA analyses (AML versus normal) were done using Oncomine engine using 5 different existing databases. Statistical significance was determined by Student t test. Significance of P values less than 0.05, 0.01 and 0.001 are shown with *, ** and *** asterisks, respectively. The combined effect of HNA with either bortezomib or As_2O_3 was analyzed by isobologram analysis using the CompuSyn software program (ComboSyn Inc, www.combosyn.com).

SUPPLEMENTARY FIGURES AND TABLES

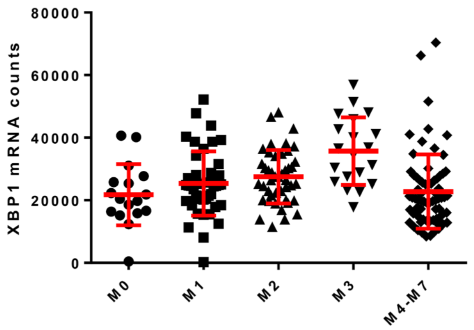

Tukey's multiple comparisons test	Mean Diff.	95% CI of diff.	Significant Summary
M0 vs. M1	-3566	-11858 to 4727	ns
M0 vs. M2	-5723	-14016 to 2569	ns
M0 vs. M3	-13931	-23511 to -4352	***
M0 vs. M4-M7	-979.8	-8832 to 6872	ns
M1 vs. M2	-2158	-8349 to 4033	ns
M1 vs. M3	-10366	-18197 to -2534	**
M1 vs. M4-M7	2586	-3001 to 8173	ns
M2 vs. M3	-8208	-16039 to -376.7	*
M2 vs. M4-M7	4744	-843.1 to 10330	ns
M3 vs. M4-M7	12952	5589 to 20314	****

Supplementary Figure 1: *XBP1* is upregulated in FAB M3 subgroup of AML patients. One-way ANOVA test of multiple comparisons of *XBP1* expression in different FAB subgroups of TCGA AML dataset. Right panel showed a summary of test result.

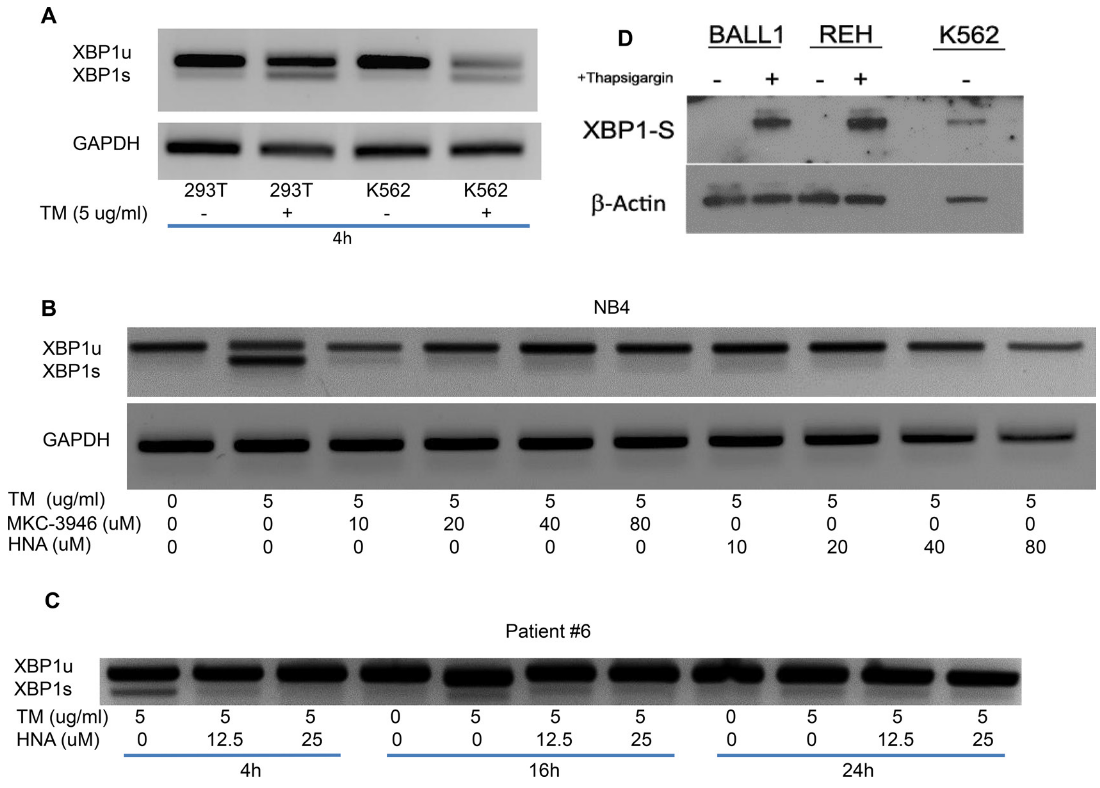

Supplementary Figure 2: IRE1 inhibitors inhibited spliced XBP1 in cells. A. Tunicamycin (TM) induced *XBP1s* expression in 293T and K562 cells. **B, C.** HNA or MKC-3946 inhibited *XBP1S* expression induced by tunicamycin (TM) in NB4 cells (B) and AML sample from patient #6. (C) Cells (10^6) were incubated with indicated concentrations of TM, STF-083010 and 2-hydroxy-1-naphthaldehyde (HNA) in 6-well plates for various durations. RNA was isolated, and RT-PCR was performed to examine *XBP1u* and *XBP1s* expression by gel electrophoresis. **D.** BALL1, REH and K562 cells were treated with or without thapsigargin (1 µM, 16 h), and expression of XBP1S were evaluated by western blotting (β-actin, loading control).

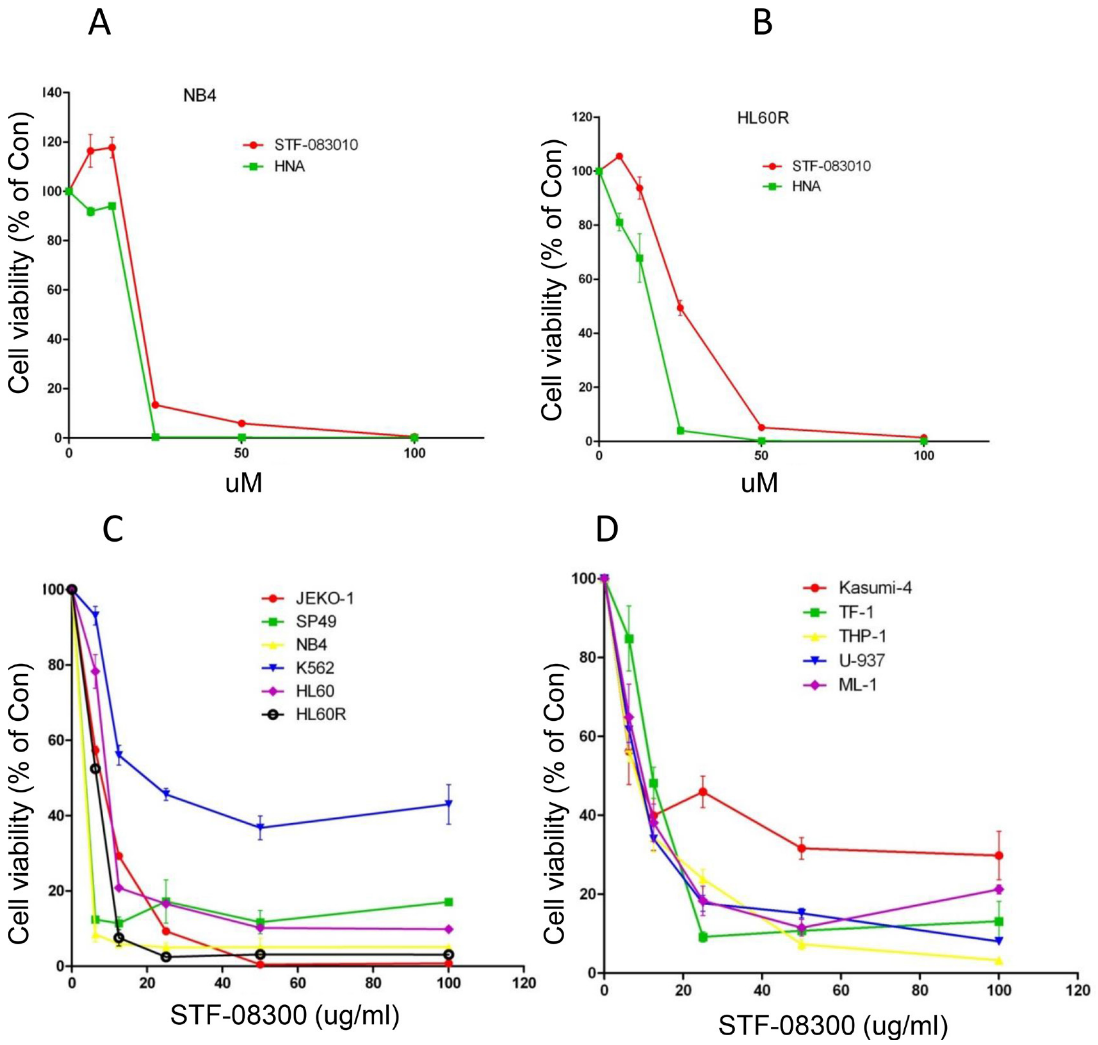

Supplementary Figure 3: IRE1 inhibitors caused cytotoxicity to AML cells Effect of IRE1 inhibitors on viability of human AML cell lines. A, B, C, D. Cells (10,000) were placed into 96-well plates, following by exposure to various concentrations of either STF-083010 or HNA. Cell viability was examined 72 h later (Material and Methods). Data represent mean ± SD, n=3.

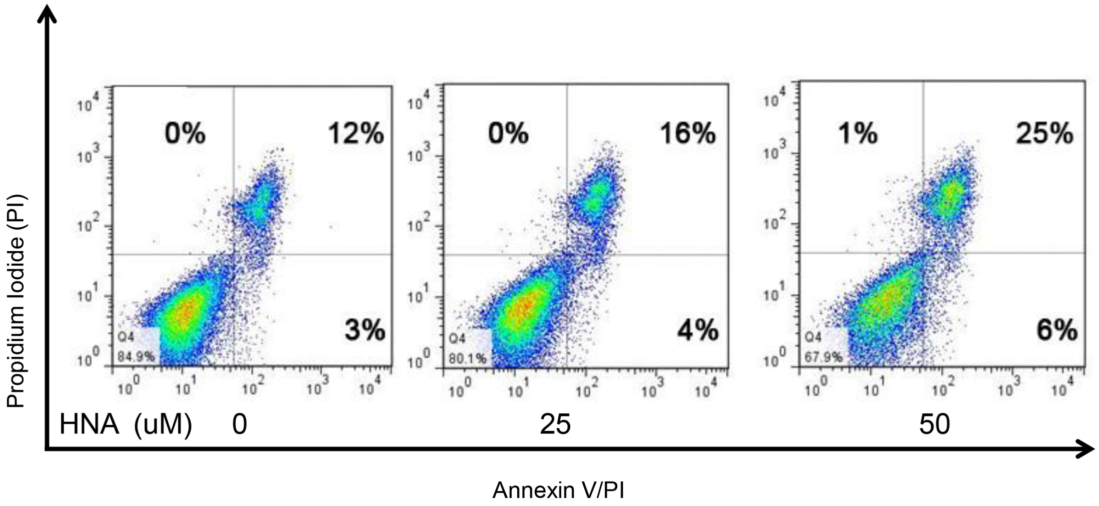

Supplementary Figure 4: HNA induced apoptosis in AML blast cells of patient #13. A. AML cells (case #13) were treated with HNA (25µM and 50µM) for 24 h and annexin/PI apoptosis assays were conducted.

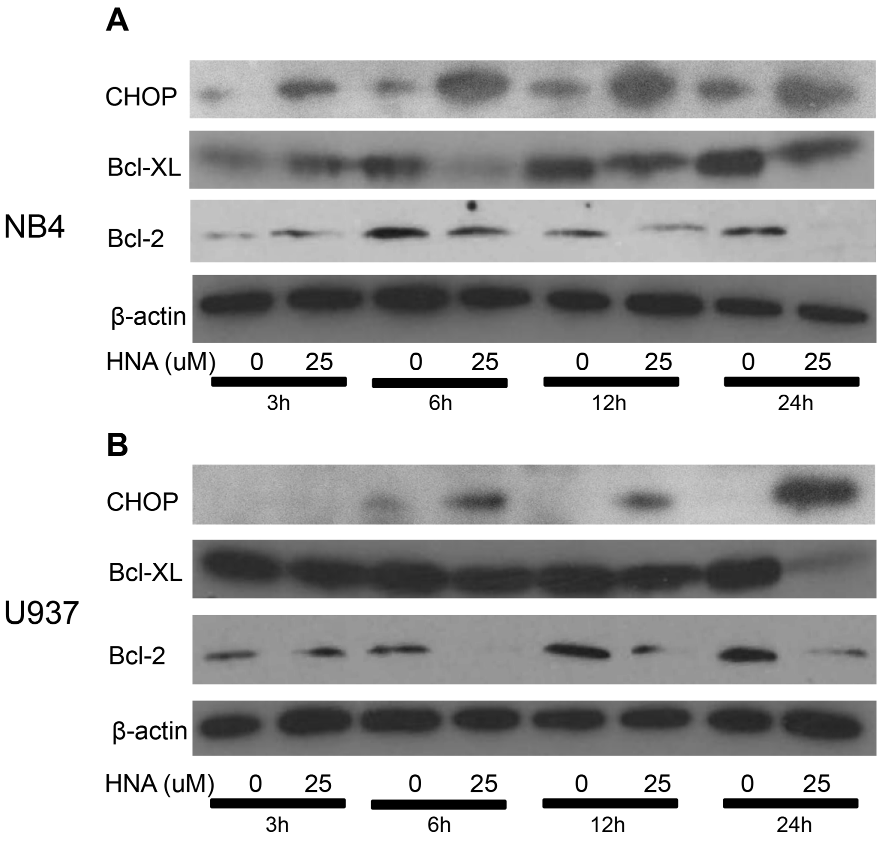

Supplementary Figure 5: Effect of HNA on expression of apoptosis-related proteins in AML cells. A, B. NB4 and U937 cells were treated with HNA (25 μM, 3-24 h), and expression of CHOP, Bcl-XL and Bcl-2 proteins were evaluated by western blotting (β-actin, loading control).

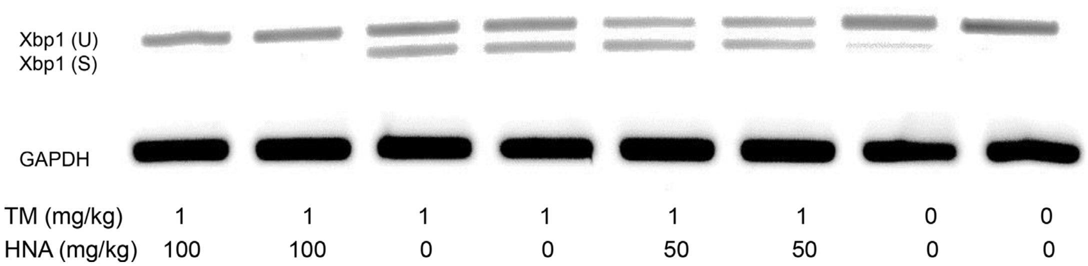

Supplementary Figure 6: HNA decreased TM-induced *XBP1S* expression *in vivo* BALB/cJ mice were treated with various concentrations of TM and HNA for 6 h. Mice were sacrificed; RNA was isolated from BM cells; and RT-PCR was performed to examine *XBP1u* and *XBP1s* expression by gel electrophoresis.

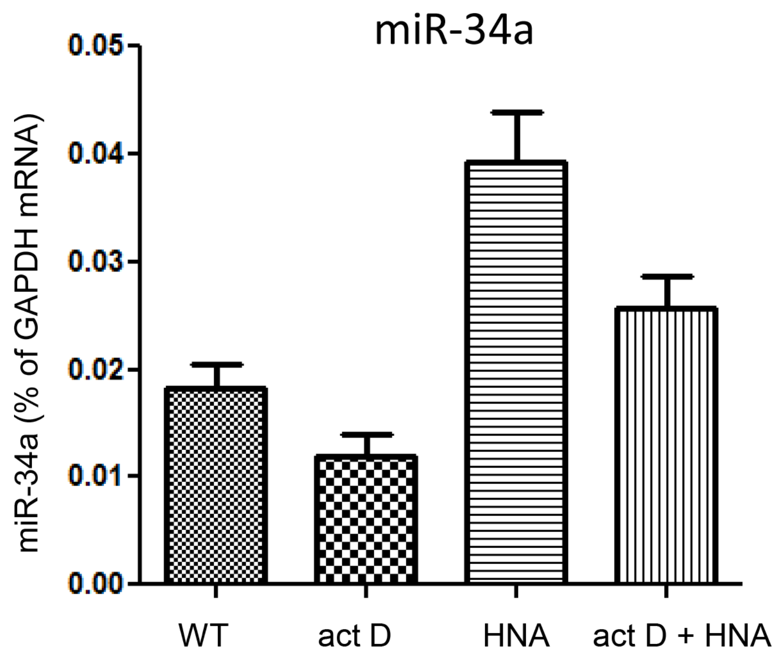

Supplementary Figure 7: Actinomycin D inhibited HNA induced miR-34a level. NB4 cells (10^6) were treated with either actinomycin D (actD, 1 µM), HNA (25 µM) or combination of both for 6h, and pre miR-34a levels were measured by QRT-PCR. Cells without HNA treatment (WT) were used as control. Relative expression of miR-34a was normalized to GAPDH mRNA expression. Data represent mean ± SD, n=3.

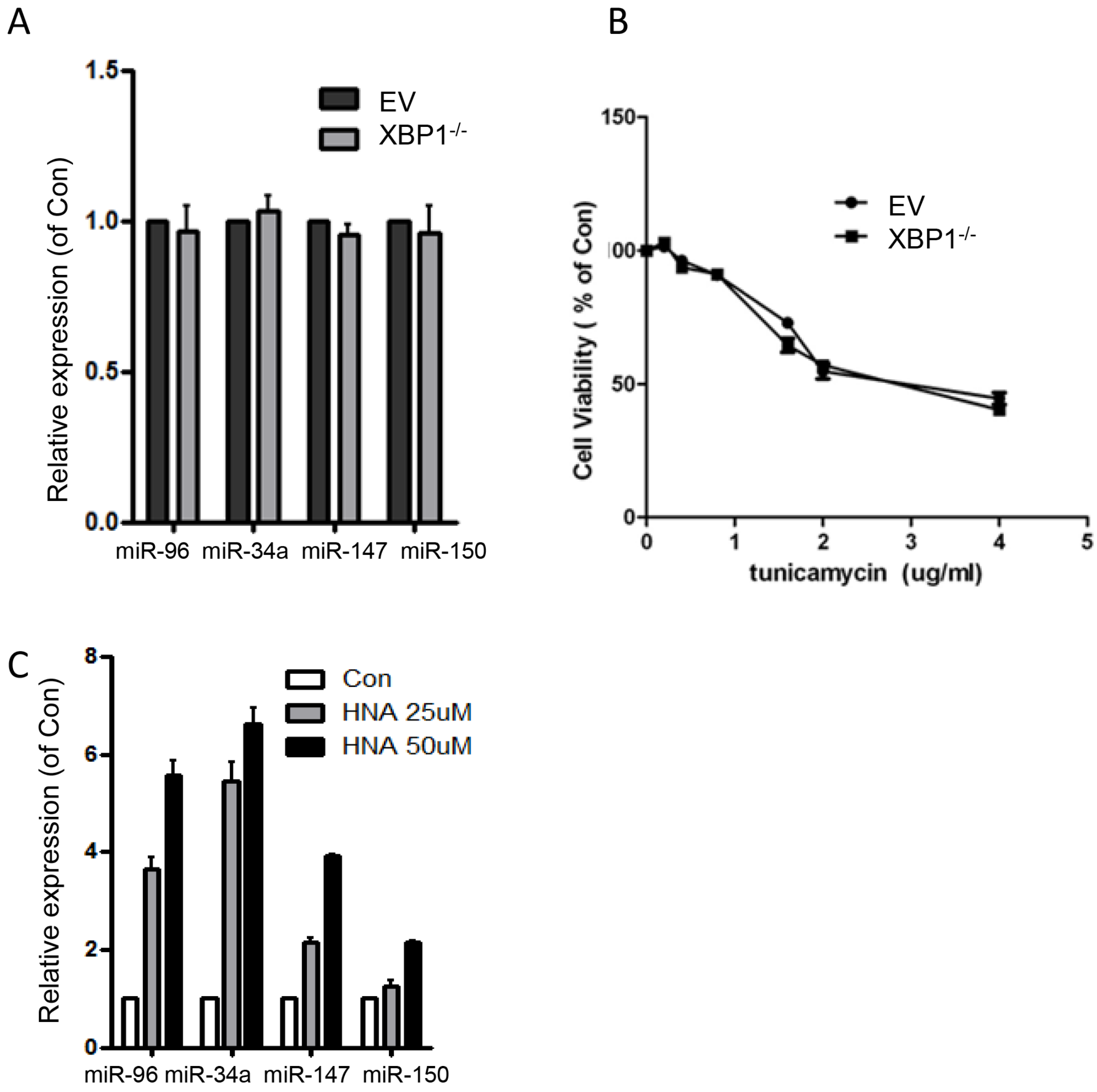

Supplementary Figure 8: Deletion of Xbp1 did not change expressions of miRNAs. A. Levels of pri-miR (-96, 34a, -147 and -150) were measured by QRT-PCR in EV (control) and Xbp1$^{-/-}$ murine bone marrow cells. Relative expression of each pri-miR was normalized to Gapdh; data represent mean ± SD, n=3. **B.** EV and Xbp1$^{-/-}$ bone marrow cells (10,000) were seeded into 96-well plates, followed by treatment with increasing concentreations of tunicamycin (72 h), and cell viability was measured (MTT assay). Data represent mean ± SD, n=3. **C.** Xbp1$^{-/-}$ bone marrow cells were treated with various concentrations of HNA (25 µM, 50 µM); and levels of pri-miRs (96, 34a, 147 and 150) were measured by QRT-PCR. Relative expression of each miR was normalized to Gapdh; data represent mean ± SD, n=3. All cells were cultured with cytokines mentioned in "Methods", and then normalized to untreated control (Con) cells.

Supplementary 1: FAB classification and defining characteristics

See Supplementary File 1

Supplementary Table 2: Primer sequences for PCR

Oligo name	Forward Sequence	Reverse sequence
	Human RT-PCR primers	
XBP1s and XBP1u	5′-CCTGGTTGCTGAAGAGGAGG-3′	5′-CCATGGGGAGATGTTCTGGAG-3′
GAPDH	5′-AACATCATCCCTGCATCCA-3’	5′-CCAGTGAGCTTCCCGTTCA-3’
	Human q-RT-PCR primers	
XBP1s	5’-TGCTGAGTCCGCAGCAGGTG-3’	5’-GCTGGCAGGCTCTGGGGAAG-3′
XBP1	5′-AGTCCGCAGCACTCAGACTA-3′	5′-AGTCAATACCGCCAGAATCC-3′
Calnexin-1	5′-TTACTGGTGCTTGGAACTGC-3′	5′-CTTGGGAGATGAAGGAGGAG-3′
GRP78	5′-GTAGCGTATGGTGCTGCTGT-3′	5′-TCATGACACCTCCCACAGTT-3′
CHOP	5′-GACTGAGGAGGAGCCAGAAC-3′	5′-ACCACTCTGTTTCCGTTTCC-3′
HERPUD1	5′-CATCTGGTGTGCAATGTGAA-3′	5′-TCCCTTTGCCTTAAACCATC-3′
DNAJB9	5′-TCATCTTTGCAATCTGCATTT-3′	5′-ACTTCATGGCCAACTTGTGA-3′
DNAJC3	5′-GACGGAGAAGATCCTTTGGA-3′	5′-ATCTAAATGGTCCGCCTGAG-3′
HEDJ	5′-GAGTCACTGGTTGGCTTTGA-3′	5′-CCTTTCTTCCATAGCTTCGC-3′
EDEM1	5′-CTGGTGGAATTTGGGATTCT-3′	5′-GTATCATTGCTCCGGAGGTT-3′
SERP1	5′-CAGTAAAGCATCCTGCCTCA-3′	5′-CAAGGCACTGTGGTATGGAC-3′
HSPA5	5′-GTAGCGTATGGTGCTGCTGT-3′	5′-TCATGACACCTCCCACAGTT-3′
pri-miR-34a	5′-CGTCACCTCTTAGGCTTGGA-3′	5′-CATTGGTGTCGTTGTGCTCT-3′
miR-34a	5′-TGGCAGTGTCTTAGCTGGTTGT	5′-Universal qPCR primers provided by Qiagen
pri-miR-144	5’-GCTGGGATATCATCATATACTG-3’	5’-CGGACTAGTACATCATCTATACTG-3’
pri-miR-96	5′ CGGAGCACCTTACCCACTT 3′	5′ CAACCCACGGCACCATTCA 3′
pri-miR-17	5′-GCTTTGGCTTTTTCCTTTTTG-3′	5′-CCTCACTGCAGTAGATGCACA-3′
pri-miR-21	5′-TTTTGTTTTGCTTGGGAGGA-3′	5′-AGCAGACAGTCAGGCAGGAT
pri-miR-150	5′-AACCCCAGCAGCAACAACTCC	5′-AGATGAGAAGAAGGCCCCAGG
SIRT-1	5′-AGAAGAACCCATGGAGGATG	5′-TCATCTCCATCAGTCCCAAA
LDNA	5′-TCTCTGTAGCAGATTTGGCAGA	5′-AAGACATCATCCTTTATTCCGTAAA
MTA2	5′-AGGGATTCGTTCAAGCTCAC	5′-GGGTCAGAGCCTTCCGTAG
CCNE2	5′-TGGCTATGCTGGAGGAAGTA	5′-GCTCTTCGGTGGTGTCATAA
CDK4	5′-ACATGTGGAGTGTTGGCTGT	5′-GCCCAATCAGGTCAAAGATT
CDK6	5′-TGCACAGTGTCACGAACAGA	5′-ACCTCGGAGAAGCTGAAACA
c-mYc	5’-AGATCCCGGAGTTGGAAAAC-3’	5’-AGCTTTTGCTCCTCTGCTTG-3’
Cyclin D1	5’-TTGTGCATCTACACTGACAAC-3’	5’-GAAGTGTTCGATGAAATCGT-3’
	Mouse RT-PCR Primers	
Xbp1s and Xbp1u	5’-AGTTAAGAACACGCTTGGGAAT-3’	5’-AAGATGTTCTGGGGAGGTGAC-3’
	Mouse q-RT-PCR primers	
Xbp1s	5’-ACATCTTCCCATGGACTCTG-3’	5’-TAGGTCCTTCTGGGTAGACC-3’
Gapdh	5’-CATGGCCTTCCGTGTTCCTA-3’	5’-CCTGCTTCACCACCTTCTTGAT-3’
