# Supplementary material for: Inhibition of IRE1α-driven pro-survival pathways is a promising therapeutic application in acute myeloid leukemia
Source: Oncotarget. 2016 Feb 25;7(14):18736–49. doi: 10.18632/oncotarget.7702 (PMC4951325; doi:10.18632/oncotarget.7702)
Supplement: Supplementary file 2 [file oncotarget-07-18736-s002.docx]

| Number | Sex | WHO/FAB and characteristics |
| --- | --- | --- |
| 1 | F | M2 normal female karyotype [46,XX] No mutation detected for FLT3 ITD, FLT3 TKD, NPM1 exon 12, or CEBPA |
| 2 | M | M2 46,XY,t(1;13)(p22;q34),del(2)(p13),t(9;21)(p21;q11),del(11) (q23),add(12)(p13)[1]/46,xx[15] No mutation detected for FLT3 ITD, FLT3 TKD, NPM1 exon 12, or CEBPA |
| 3 | M | M1 FLT3+  Insufficient for cytogenetic analysis due to poor quality dividing cells |
| 4 | M | M4 inv(16)(p13.1q22) 47,XY,+8,inv(16)(p13.1q22)[15] by conventional cytogenetic analysis No mutation detected for FLT3 ITD, FLT3 TKD, NPM1 exon 12, or CEBPA |
| 5 | F | M5 complex female karyotype with a hyperdiploid abnormal clone with t(6;11) involving MLL rearrangement confirmed also by fish: 49,XX,+3,t(6;11)(q27;q23),+der(6)t(6;11),+8[20].ish t(6;11)(3'MLL+; 5'MLL+,3'MLL-), der(6)(3'MLL+)[5]  No mutation detected for FLT3 ITD, FLT3 TKD, NPM1 exon 12, or CEBPA |
| 6 | M | M4 normal female karyotype [46,XX] No mutation detected for FLT3 ITD, FLT3 TKD, NPM1 exon 12, or CEBPA |
| 7 | M | M4 Normal male karyotype by conventional cytogenetic analysis Negative for FLT3 TKD, Positive for FLT3 ITD and NPM1 mutations; and CEBPA mutations |
| 8 | F | M2 Trisomy 13 in 85% of cells on karyotype analysis (47,XX,+13[17]/46,XX[3]) No mutation detected for FLT3 ITD, FLT3 TKD, NPM1 exon 12, or CEBPA |
| 9 | F | M1 normal female karyotype 46,XX[20] FLT3+ , npm1- |
| 10 | M | M0 47,XY,+13,i(13)(q10)x2[8]/46,XY[12] No mutation detected for FLT3 ITD, FLT3 TKD, NPM1 exon 12, or CEBPA |
| 11 | F | M1 46,XX,del(5)(q22q31)[11].46,XX[3] No mutation detected for FLT3 ITD, FLT3 TKD, NPM1 exon 12, or CEBPA |
| 12 | F | Acute myelogenous leukemia with monocytic differentiation, therapy-related   46,XX,t(9;11)(p21;q23)[20] MLL+ |
| 13 | F | AML with myelodysplasia-related changes  normal female karyotype 46,XX[20] No mutation detected for FLT3 ITD, FLT3 TKD, NPM1 exon 12, or CEBPA |
| 14 | M | M2 8;21 translocation t(8;21)(q22;q22); RUNX1-RUNX1T1  45,X,-Y,t(8;21)(q22;q22)[7]/46,XY[1] KIT D816V: Positive No mutation detected for FLT3 ITD, FLT3 TKD, NPM1 exon 12, or CEBPA |
| 15 | M | M4 Normal male karyotype in limited study.   FLT3 Internal Tandem Duplication (ITD)- FLT3 TKD+ Positive for a NPM1 + CEBPA+ |
| 16 | M | M4 44,XY,del(3)(q12),-5,add(6)(q21),-7,add(12)(p13),add(15)(q26), add(16)(p13.3),-21,-22,+mar1,+mar2[8]/44,idem,-1,+3,add(9)(q34), der(11)t(1;11)(p13;p11.2),+22,-mar2[3]/46,XY[7] No mutation detected for FLT3 ITD, FLT3 TKD, NPM1 exon 12, or CEBPA |
| 17 | M | M4 AML with t(10;11)(p12;q23.3) involving MLL (11q23) with most likely MLLT10 gene at 10p12;MLLT10-MLL  MLL rearrangement detected by FISH 46,XX,dup(5)(?q31q31),der(10)inv(11)(q13.1q23.3)t(10;11)(p12;q23.3), der(11)t(10;11)[20].ish dup(5)(wcp5+),der(10)(KMT2A+, 5’KMT2A sep 3’KMT2A), der(11)(KMT2A-)[3] Negative for FLT3, NPM1 and CEBPA mutations |
| 18 | M | M5 45,Y,del(X)(p11.4),del(5)(q13q33),-18[16]/46,XY,t(2;5)(p?23;q?25)[4]  Deletion of 5q detected in 81% of cells by FISH No mutation detected for FLT3 ITD, FLT3 TKD, NPM1 exon 12, or CEBPA |
| 19 | F | M2 46,XX[20] FLT3+ , npm1- |
| 20 | F | M2 Trisomy 8 in 80% of cells [47,XX,+8] |
| 21 | F | Therapy related AML with MDS changes "Monosomal karyotype" by conventional cytogenetic analysis: 40,XX,del(5)(q13q35),-6, -10,add(11)(p15),-13,add(14)(p11.1),add (15)(p11.1),add(16)(p13.1),-17,-17,-20, add(21)(q21),der(21)add(21) (p11.2)del(21)(q22)[4]/46,XX[3] -Deletion of 5q detected in 85% of cells by FISH -Negative for FLT3 (ITD and TKD), NPM1 and CEBPA mutations |
| 22 | M | M2 FLT3- , npm1- 46,XY[20] No mutation detected for FLT3 ITD, FLT3 TKD, NPM1 exon 12, or CEBPA |
| 23 | M | M2 46,XY,i(21)(q10)[4]/46,sl,del(11)(p?13p?15)[10]/46,XY[6] No mutation detected for FLT3 ITD, FLT3 TKD, NPM1 exon 12, or CEBPA |
| 24 | F | M2 +Normal female karyotype No mutation detected for FLT3 ITD, FLT3 TKD, NPM1 exon 12, or CEBPA |
|  |  |  |
|  |  | AML with minimal differentiation (M0) AML without maturation (M1) AML with maturation (M2) Acute myelomonocytic leukemia (M4) Acute monocytic leukemia (M5) Acute erythroid leukemia (M6) Acute megakaryoblastic leukemia (M7) |
